# A flexible piezoresistive strain sensor based on laser scribed graphene oxide on polydimethylsiloxane

**DOI:** 10.1038/s41598-022-08801-0

**Published:** 2022-03-22

**Authors:** Maham Iqra, Furqan Anwar, Rahim Jan, Mohammad Ali Mohammad

**Affiliations:** School of Chemical and Materials Engineering, National University of Sciences and Technology, Islamabad, 44000 Pakistan

**Keywords:** Materials science, Nanoscience and technology

## Abstract

Flexible strain sensors are an important constituent in soft robotics, health care devices, and in the defence industry. Strain sensors are characterized by their sensitivity (gauge factor-GF) and sensing range. In flexible strain sensors, simultaneously achieving consistency and high sensitivity has always been challenging. A number of materials and their derivatives have been explored to achieve balanced sensitivity with respect to sensing range with limited results. In this work, a low-cost flexible piezoresistive strain sensor has been developed using reduced graphene oxide (rGO) on polydimethylsiloxane (PDMS). The reduction has been performed using laser scribing, which enables the fabrication of arbitrary structures. After lead-out, the devices were again sandwiched in a layer of PDMS to secure the structures before performing their testing using a locally developed testing rig. Compared to previously reported graphene strain sensors, the devices fabricated in this work show relatively high GF with respect to sensing range. The GF calculated for stretching, bending and torsion was 12.1, 3.5, and 90.3 respectively, for the strain range of 0–140%, 0–130%, and 0–11.1%. A hand test was performed for the detection of joint movement. Change of resistance has been observed indicating muscle motion.

## Introduction

Flexible strain sensor developments have rapidly picked up in the last few years due to their low-cost fabrication and diverse applications which can rise to an entirely new industry. Flexible strain sensors have a different market in comparison to conventional strain sensors due to their flexibility and biocompatibility. In the healthcare industry, these devices show very good potential in real-time monitoring of heart rate^1^, muscle motion^2^, and asthma detection^3^ etc. The sensor works for soft robotics^4^ and sports performance monitoring^5^ as well.

The strain sensor consists of an electrically active material that is fabricated on the substrate of choice, thus used to sense the strain by electrical, resistance, capacitance or inductance change. Strain represents the change in length or angle under applied stress. This includes stretching, compression, bending and torsion. The sensitivity of these devices can be appraised by gauge factor (GF), which can be expressed as the ratio of relative change of electrical resistance R to the mechanical strain ε:

1 $$\mathrm{GF}=\frac{\left(\mathrm{\Delta R/R}\right)}{\large\varepsilon }$$

Reduced Graphene Oxide (rGO) has enhanced electrical properties due to the C–C bond recovery^6^ after laser reduction. Laser is considered to be a precise writing technique that helps in making micro size patterns in the shape of choice. However, laser irradiation also has been found to enable the conversion of a non-conductive material (GO) into a conductive material (rGO)^7,8^ by changing the chemistry of the material at high temperature^9^. Photo-thermal reduction^10^ causes the material to expand. This results in the formation of a multilayer structure which is understood to be composed of overlapping flakes. These flakes take an important part in the piezoresistive behaviour, as the resistance changes with the overlapping area of the flakes^11^. When combining these rGO flakes with polydimethylsiloxane (PDMS) polymer, we obtain both electrical and flexible properties in the same device which enables the coverage of a wide application area. Due to the excellent mechanical and electrical properties, GO becomes the perfect candidate for strain sensing and the ideal outcome is to achieve high sensitivity, reliability, mass production and good sensing range.

Piezoresistive strain sensors are the most extensively studied strain sensors^11–14^. Furthermore, sensors made of Carbon-based materials and their composites also show piezoresistive behaviour^15^ and have very good electrical, chemical, mechanical and optical properties^16–19^. Structural modifications and chemical confinement of two-dimensional materials like graphene, GO and rGO are widely exploited to fabricate strain sensors. Flexible strain sensors have an extensive application range including smart clothing made by rGO coated elastic fibres which show GF of 8.8 below 5% sensing range^20^. Recent studies show that the combination of graphene with Molybdenum-Carbide^21^ results in a GF of 73(tension) and 43(compression) for a lower sensing range of 0.07 to 0.25%. Paper-based strain sensor was also studied by casting graphene flakes onto mulberry paper attaining the sensitivity of 3.82 at 0.587%^22^. Strain range increases to 800% when infiltrated with elastic bands but GF reduces to 36^23^. The common theme witnessed in these studies is that the material only provides one of them—either good GF or high strain tolerance. This problem restricts the application area, therefore achieving high sensitivity for the specific strain range is still a challenge. In earlier studies^11^, GO was applied on PI and reduced to make a four degree of freedom (4-DOF) strain sensor yielding a GF of 54.2 at a sensing range of 0.003%. To achieve six degrees of freedom (6-DOF), GO was applied to PDMS polymer material. Despite having lots of literature on 3D materials like graphene foam on PDMS^24^, the effect of strain on 2D rGO/PDMS is hardly studied due to the hydrophobic property of PDMS which prohibits the GO adhesion with the PDMS layer.

The current research reports the fabrication of thin-film-based strain sensor. This thin-film strain sensor consists of laser scribed rGO sandwiched between PDMS layers. A systematic study was conducted to address the issue of GO/PDMS layer adhesion. The laser scribed rGO/PDMS structure not only exhibits 6-DOF but also shows an excellent GF. This fabrication method promises low-cost mass production. A strain sensor was further attached with the human hand and finger for muscle motion detection. A visible change in resistance has been observed after stretching and compression.

## Experimental details

**Figure 1:**
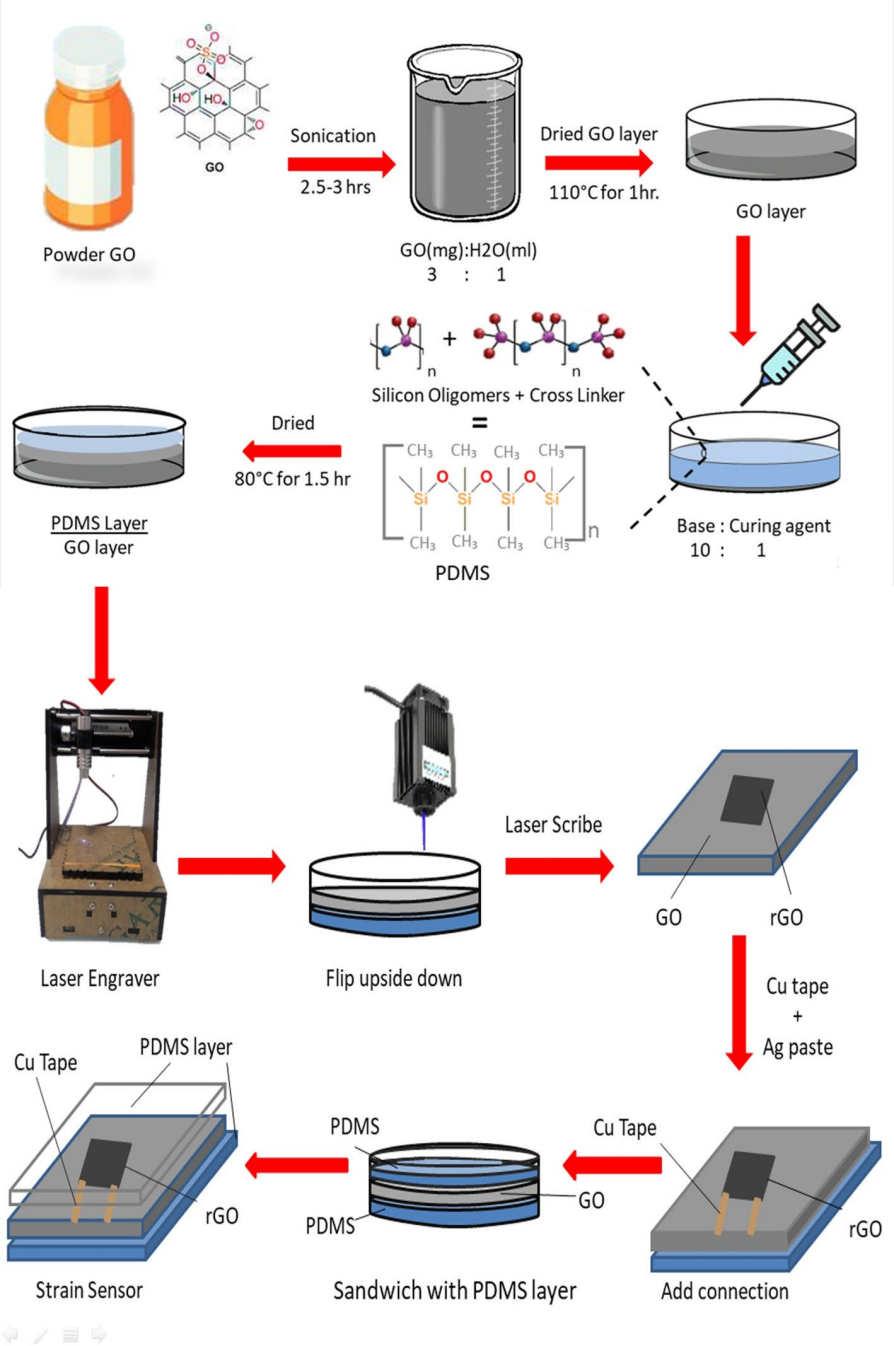
Schematic illustration of the fabrication of a strain sensor composed of laser scribed rGO and PDMS. The process includes synthesis of GO, adhesion with PDMS, laser patterning (reduction) of rGO/PDMS stack, lead-out, and sandwiching the structure in PDMS. (Chemical structures were drawn using Chem Draw by PerkinElmers Informatics and Images were drawn using Adobe Illustrator and Microsoft PowerPoint drawing tools and assembling was done using Microsoft PowerPoint while some images were taken from online image gallery^27–29^).

The process flow of fabricating a strain sensor is visualized in Fig. 1. Modified Hummers method^25^ was used to synthesize GO. The solution of GO was prepared in water with a 3:1 ratio and sonicated for about 2.5 h to agitate GO particles in the water. GO solution was then drop cast and dried approximately for 1 h at 110 °C in a petri dish to make a thin film. PDMS Sylgard®_184 (Dow Corning Corporation) is drop cast after combining the two-part sets of cross-linker (curing agent) and pre-polymer (base) in the ratio of 1:10 respectively. This PDMS layer is then dried in an oven for 120 min at 80 °C. It may be noted that the GO layer was dried in a petri dish first and then the PDMS layer was poured on top of the GO layer. The resulting stack was flipped upside down after drying in preparation for laser irradiation of the GO. The mechanism behind this specific order of preparing the GO and PDMS layers is that liquid PDMS partially penetrates inside the porous structure of the GO that results in achieving better layer adhesion^26^. After flipping, the rGO/PDMS stack was patterned using a laser. The laser scribing is shown in Supplementary Fig S1a whereby a predefined pattern is drawn. A wavelength of 440 to 445 nm laser scriber (as shown in Supplementary Fig S1c is operated by using software through a laptop as shown in Supplementary Fig S1b. A pixelated 1 × 1 cm square shape pattern is drawn on Adobe Illustrator and uploaded on the laser software. GO was scribed using 50 to 100 mW laser power as shown in Supplementary Fig S1d. This image was then patterned twice one pixel apart on top of the first one (0° offset). The addition of this step is to scribe between the already scribed lines for better conversion of GO into rGO. Following the laser reduction, the connections are added by using copper tape and silver paste. The device is then sandwiched with PDMS by using a drop-casting and drying step mentioned earlier. The addition of this step is to avoid buckling and misalignment after applying strain.

**Figure 2:**
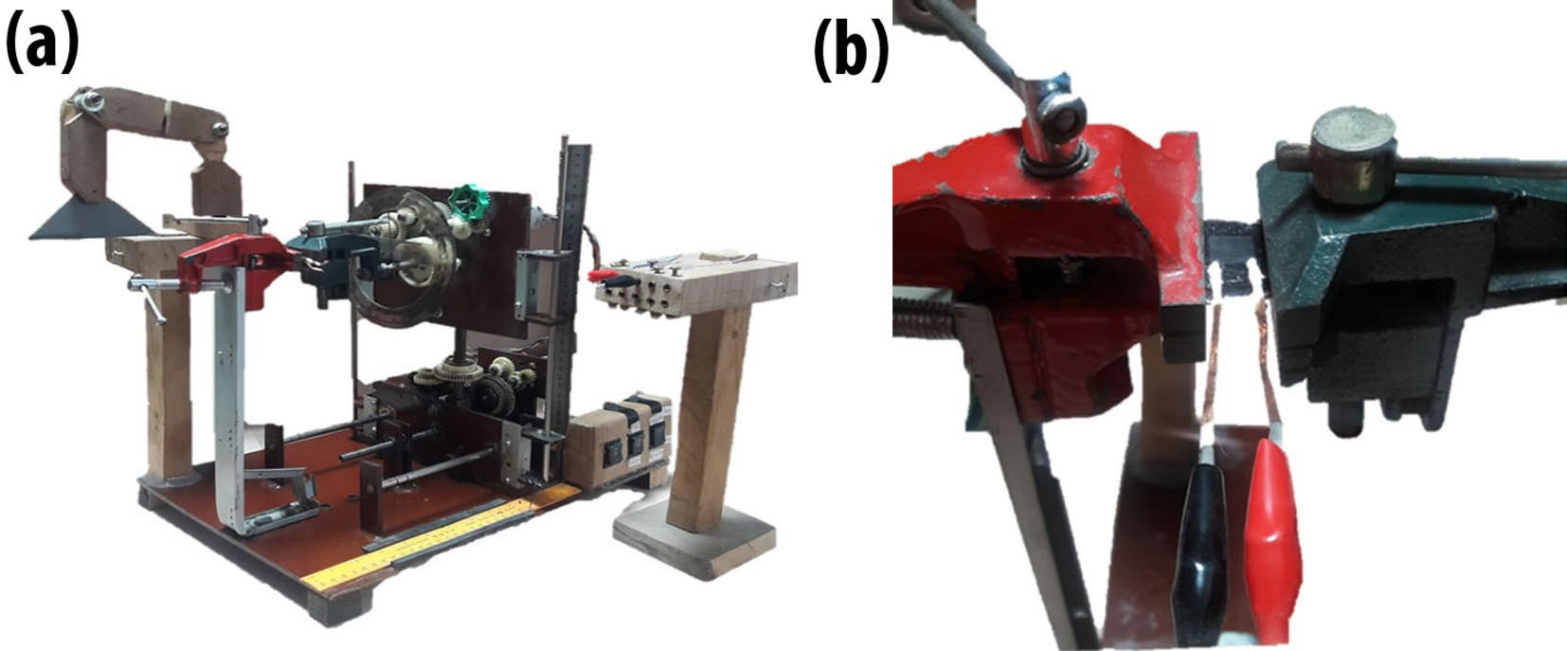
(**a**) Locally developed electrically actuated testing rig is proficient for applying five types of strain for thin films. It works for stretching, compression, bending (upside, downwards), and torsion (clockwise, anti-clockwise). (**b**) The clamp structure contains jaws to hold the sensor in place and to apply strain. One of the jaws is fixed, while the other is electrically actuated.

The strain was applied to the sensor by using a locally developed testing rig. The testing rig is actuated using a DC motor that moves the gears assembly connected to the set of claws. This device is capable of applying six types of strain to the thin films including stretching and compression, bending (upward, downward), and torsion (clockwise, anti-clockwise). The sensor was held in between the two claws of the testing rig, while simultaneously being connected with the digital multi-meter as shown in Fig. 2. The output was acquired using a digital multi-meter as resistance change and then visualized using software.

### Ethical approval of research

The research was permitted by the NUST Institutional Review Board (IRB), which is the authorized committee of ethical review of all research conducted at the university. The research was conducted according to acceptable standards as defined in NUST ethical review policy.

### Informed consent

The test (Fig. 6) was conducted using the hand of one of the co-authors (MI). MI fully consented to the testing and she herself volunteered to the testing with her own free will. Moreover, no harm to the body was witnessed during the experiments.

## Results and discussion

### Material characterization

**Figure 3:**
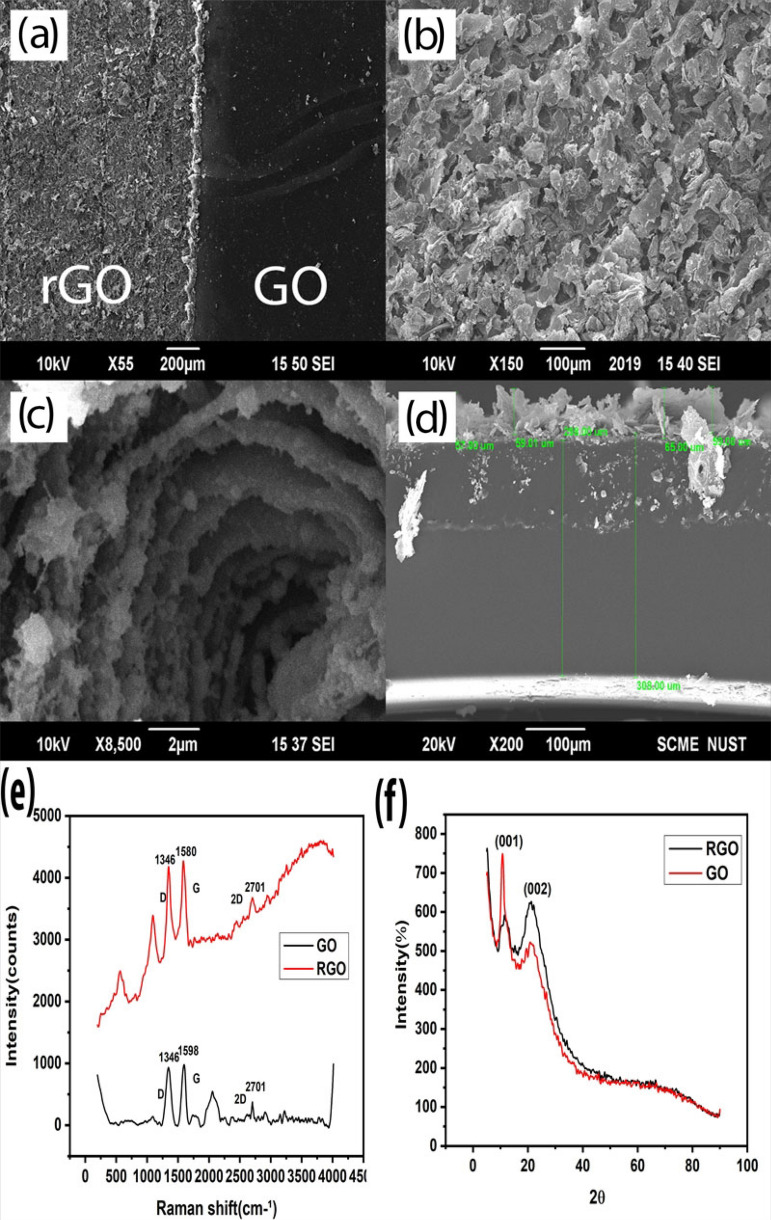
Characterization of GO and laser scribed rGO. Morphology comparison of (**a**) SEM images depicting the difference between the surface structure of GO and rGO. (**b**) The porous structure of rGO after reduction. (**c**) rGO flakes sharing their surface area with each other helps in sensing strain. (**d**) The thickness of rGO increases with respect to GO after reduction. (**e**) RAMAN spectroscopy of GO and rGO depicts that the reduction has taken place. (**f**) A sharp peak of XRD Diffraction at 10.75° confirms GO and after the reduction, another peak at 22.1° confirms rGO structure.

SEM was performed to analyse the surface of rGO after laser reduction. In Fig. 3 we can clearly see the difference in surface morphology: Panel (a) depicts the difference between surface morphology of GO (smooth surface) and rGO (rough surface). The rough porous surface in Panel (b) is rGO. The high temperature of the laser causes the GO structure to inflate by removing oxygen-containing functional groups in the form of gases. These gases intercalate between the layers of graphene oxide leaving behind the swelled flaky structure of rGO. Panel (c) shows these loosely packed flakes^30^ that aid in sensing strain^9^. Panel (d) shows that the thickness of rGO increases in the range of 57 to 65 µm compared to GO.

As depicted in Fig. 3e, RAMAN spectroscopy (BWS415-532S, USA) was performed to analyse the material and the conversion of GO into rGO. The D and G peak clearly shows the conversion of GO into rGO. The D peak indicates the presence of defect structure in the carbon lattice due to functional groups in the sp^3^ hybridized structure. The shift in G peak indicates the double bond of sp^2^ hybridized structure of GO^31^. It also indicates the addition of material as doping or subtraction of material as reduction. The left shift from 1599 cm^−1^ to 1587 cm^−1^ specifies the reduction process, while the slight decrease in the D peak of rGO tells us that some of the C–C structure has been recovered. The presence of 2D peak indicates that the stacked multilayer flaky structure has been created^9^.

X-ray Diffraction results of GO and rGO were performed as shown in panel (f) of Fig. 3. The Cu source having wavelength 1.54 Ǻ of STOE θ–θ XRD diffractometer is used to determine the structure. The diffraction graph of XRD shows that the diffraction peak of GO at 2Ө (10.75°) by (001) plane is due to oxygen-containing functional groups. The sharp peak is indicative of the structure of GO with the *d* spacing of 8.22 Ǻ similar to the literature^32^. When converted from GO to rGO, this peak reduces its intensity which depicts the removal of functional groups. Another broad peak is observed at 22.1° with the orientation at (002). Broadening confirms the reduction of GO and poorly arranged sheets along the stacking direction. The *d*-spacing reduces to 4.02 Ǻ^33^ due to the removal of functional groups^34^. This reduction specifies single layer or few-layer graphene structure^33^.

The *d*-spacing is actually the distance between adjacent GO sheets. GO has higher *d*-spacing than rGO as shown in Table 1 due to the presence of functional groups^35^. This can be calculated by using Bragg’s law.

**Table 1:** *D-*spacing difference of GO and RGO.

	Ɵ	*d* spacing
GO	5.375	8.22 Ǻ
rGO	11.05	4.02 Ǻ

### Device characterization

**Figure 4:**
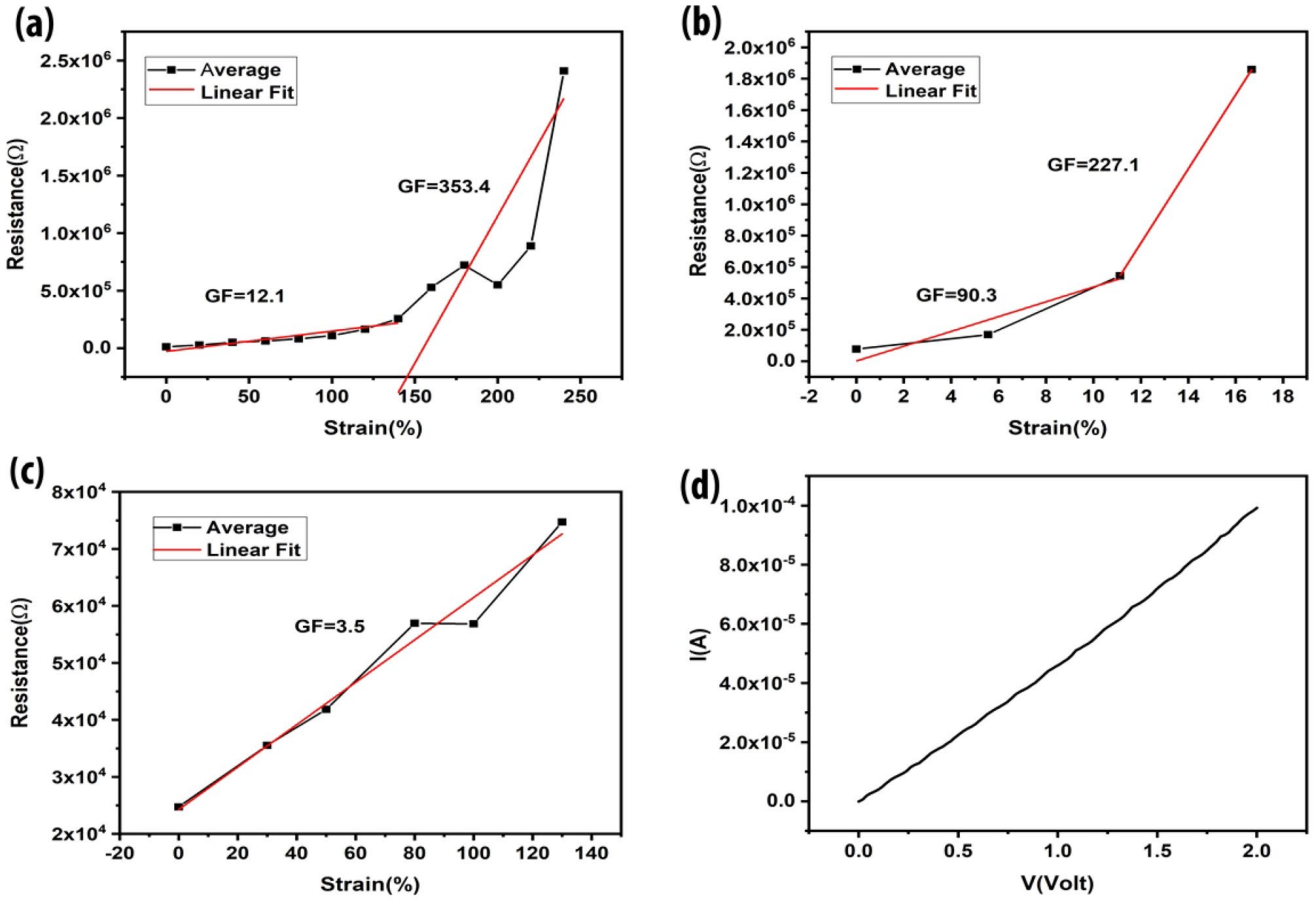
Device characterization of Stretching, Bending and Torsion. Graphs show the resistance change vs. strain percent. (**a**) An average behaviour of the device shows two ranges separated by linear fit after stretching. (**b**) Torsion also shows two sensing ranges: GF for lower range is 90 and higher range shows GF of 227. (**c**) Bending shows a very linear increase in resistance throughout the sensing range while connection breaks suddenly when the sensing limit was reached. (**d**) Current vs voltage graph without any load applied to the sensor.

After fabrication, the sensor was tested by applying different kinds of strain. The different modes of strain is shown in Supplementary Fig S2. A stretching test was performed on the sensor. Change in resistance vs. strain graph has been plotted. Resistance change has been measured with respect to length after every 2 mm scale. Change in resistance of the device works on the mechanism of overlapping areas of rGO sheets^11^. Upon stretching, the overlapping area of sheets decreases and this causes the resistance to increase. Eight successful runs of different strain sensors were performed with the same fabrication method as shown in Supplementary Fig S3 to get the average behaviour of the device as shown in Fig. 4a. Stretching the device shows a linear change from 0 to 140% strain with the gauge factor of 12.12. After further stretching to 240% strain with a gauge factor of 353.38, this sensor shows a sudden increase in the resistance which may be attributed to the cracks produced in the rGO layer. Upon maximum strain, these cracks widen and breakage in conductivity paths causes a drastic increase in resistance. This shows us that this sensor works perfectly till 140% strain. Linear behaviour of the sensor device can also be seen in Fig. 4a for both regions separately.

Torsion tests were performed by twisting the strain sensor at an angle by using a testing rig. This twisting mechanism changes the rGO sheet’s overlapping area which reduces the conductance and increases resistance. Measurements were recorded after every 5° angle. Resistance vs. strain percent average graph can be seen in Fig. 4b. Twelve successful tests of different devices prepared by the same fabrication method have been taken as shown in Supplementary Fig S4. Linear fitting for these two regions is conducted separately. The average behaviour shows a linear increase from 0 to 11.12% strain with a GF of 90.27. The resistance increases suddenly by increasing the strain to 16%, with a corresponding GF of 227.13. This drastic increase in resistance caused by excessive twisting after exceeding the certain sensing (twisting) limit, produces cracks in the structure of rGO. This structure ruptures the conductive pathways and increases resistance till there is no connection left. Therefore, we may conclude that the sensor works best for the range of 0 to 11.12% strain for torsion.

Bending tests were also performed on the testing rig. Seven sensors were tested against bending shown in Supplementary Fig S5. Average and linear strain behaviour can be seen in Fig. 4c. Measurements were taken after every 2 mm bending. This testing shows very similar behaviour by each device with minute resistance changes from each other. Sensor shows results for a maximum of 130% of strain which indicates that it has a very high bending range but the change in resistance is very linear resulting in a GF of 3.5. Bending doesn’t seem to show sudden resistance change because the connection breaks immediately after normal sensor behaviour.

A CV graph was obtained to measure the working range of the sensor. Figure 4d shows that the working potential window of the sensor was calculated to be 2 V.

### Mechanism of strain sensing

The laser tends to remove the functional groups at high temperature simultaneously causing inflation of the GO material. The functional groups react with the oxygen in air and convert into gases that intercalate into the structure of reduced graphene oxide. This behaviour causes the formation of islands (flakes) of rGO sheets. These flakes share overlapping area with each other as shown in the SEM. The change in overlapping area of flakes causes the resistance to change under applied strain.

**Figure 5:**
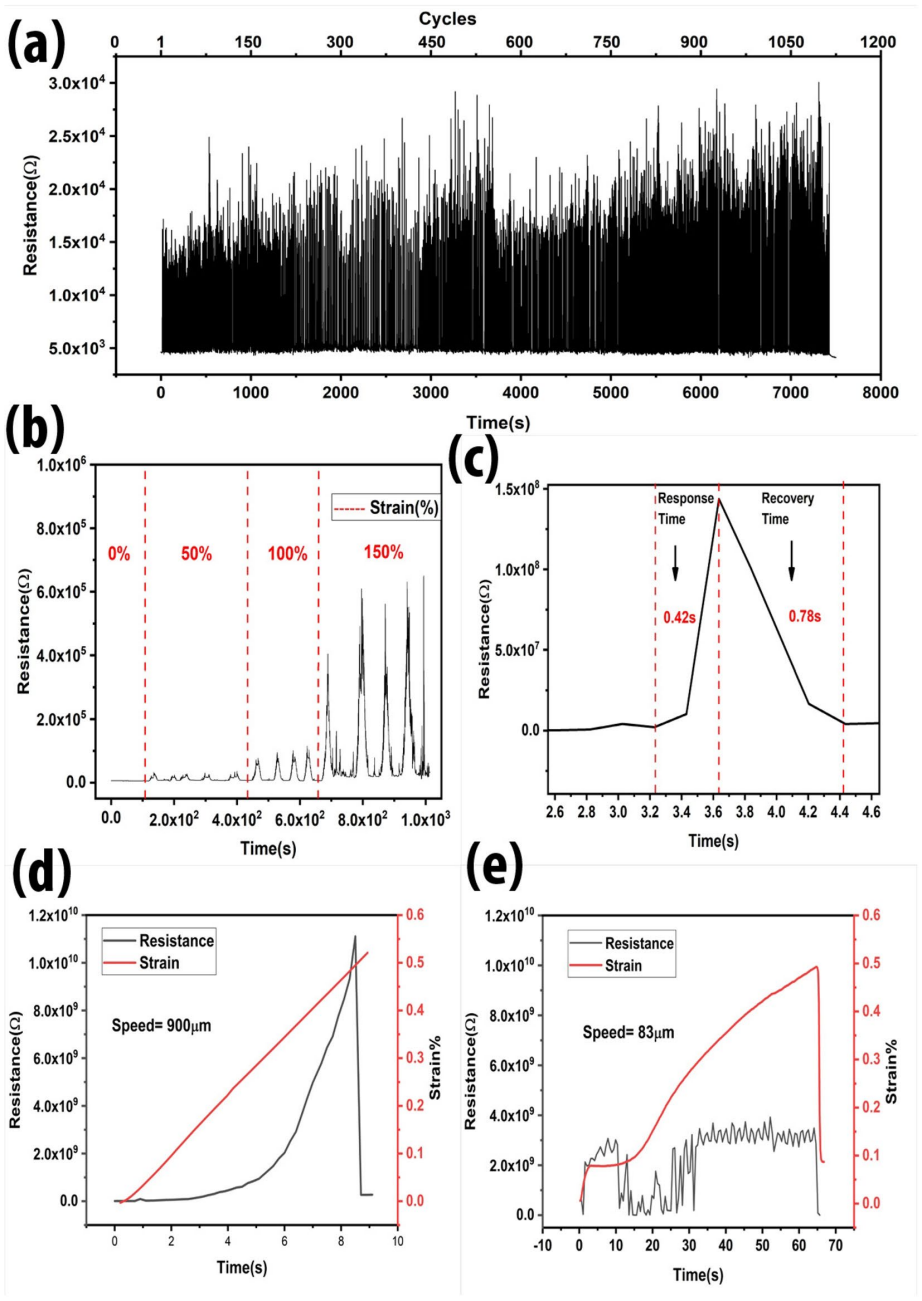
Sensor stability tests including (**a**) cyclic performance of strain sensor showing more than 1000 cycles at the strain range of 80 to 120%. (**b**) Sensor performance during different strain, for 0, 50%, 100%, and 150% strain. Resistance increases gradually after increasing the strain percentage. (**c**) Response time and recovery time was calculated as ~42 ms and ~78 ms, respectively. (**d**) Strain speed of 900 μm/s and (**e**) 83 μm/s was applied to the strain sensor to observe the sensor behaviour.

Stability tests of rGO-PDMS were also performed. Cyclic performance of this sensor shows stable sensitivity for > 1000 cycles for the strain range of 80% to 120% as demonstrated in the Fig. 5a. The changes in the intensities is due to the different strain range. While the initial resistance does remain similar as originally started even after 1000 cycles which specifies the excellent hysteresis performance of the rGO-PDMS strain sensor. While the Fig. 5b shows the effect of the different stretching ranges applied to the strain sensor. Stretching strain of 50%, 100%, and 150% was applied simultaneously by using the testing rig. It can be seen that the difference in resistance intensities of the device at 0% resistance was near zero, while at 50% strain resistance increases to 3×${10}^{4}.$ While increasing the strain to 100%, the resistance value increases up to 1×${10}^{5}$. Further stretching till 150% increases the resistance to around 6 × ${10}^{5}$. Figure 5c shows the response time was measured to be ~42 ms while the recovery time of ~78 ms was observed after a complete cycle of loading and unloading. Response time was observed to be affected by strain speed. The Fig. 5d,e shows the difference of resistance change by different strain speeds applied by tensile testing device. This tensile device has a measuring range of 1 μm to 900 μm. When the sensor is placed in a tensile testing device at its original starting state, the device has certain misalignment due to bending. When the stretching starts, the device takes a few seconds to straighten out and then the stretching begins to takes place from 4 s to around 9 s. When the resistance graph was plotted with respect to time and strain, it can be observed that the resistance is affected by the speed of the applied strain. At high speed, the resistance reaches to its maximum till 1 × 10^10^ Ω while at low speed, the resistance change is around 3 × 10^9^ Ω. There is also a difference of response time at high (900 μm/s) and low (83 μm/s) speed of the strain applied.

**Figure 6:**
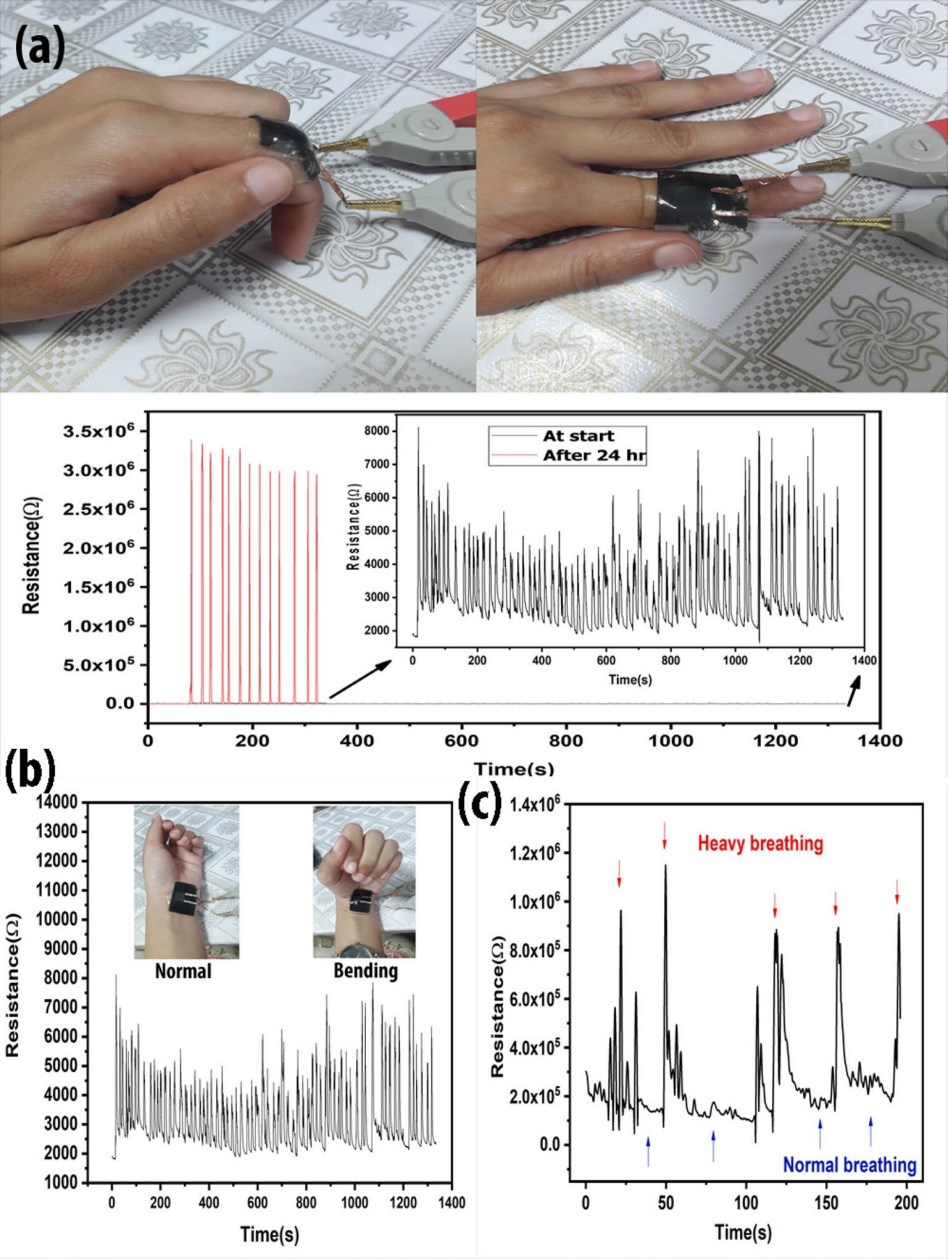
Application of the strain sensor (**a**) Sensor was applied to human finger for 24 h, stretching tests were performed at the start and end of the experiment by bending the finger at an angle between 110° to 120°. (**b**) Sensor was applied to the human hand for muscle motion detection for 24 h while measuring the stretching and compression movement of hand. (**c**) The resistance fluctuates by pulse rate resulting after a change in breathing.

Human skin is very sensitive and also 90% flexible. The material that is attached to the skin should also have enough flexibility. PDMS is a bio-compatible material, currently used to make eye lenses. A very thin layer of PDMS is capable of sticking to the skin. Here we use few mm thin layer of PDMS, which requires double sided tape to attach the strain sensor to the skin. The sensor was attached to human finger for 24 h with normal house hold routine. In Fig. 6a, the graph demonstrates the linear resistance behaviour throughout the testing. Two graphs show the resistance behaviour at the start of the experiment and at the end of the experiment after 24 h. A slight difference in the intensity of the resistance is due to the slightly different bending angle while moving the finger. The change in resistance is linear but the high increase in resistance is due to the copper tape that oxidized at much faster rate during routine house chores than normal.

This strain sensor can also be used for muscle motion detection. Figure 6b shows a strain sensor attached to the joint of the human wrist for muscle motion detection. Bending moment applies bending strain on the sensor as shown in the Supplementary video V1. The graph is visible in Fig. 6b. This sensor also shows the visible difference in normal and heavy breathing as demonstrated in Fig. 6c. Heavy breathing causes the pulse to increase and the sensor that is attached to the human wrist was capable of measuring that change. Necessary precautions were done in conducting the experimentation on the hand of the participant according to the accepted protocols. A similar change in resistance can be seen after bending phenomenon occurred by moving the hand. The time vs. resistance was formulated which is visible in Fig. 6b.

An array of four sensors were also fabricated to measure the pressure distribution as shown in Supplementary Fig S6. Resistance change was confirmed after applying pressure to the sensor array. In future work, array structures will be studied in detail.

Comparison between sensitivity and sensing range of graphene-based strain sensors with this work can be seen in Table 2. Observation from recent literature shows that graphene and its derivatives have low sensing range except when used with Poly(Styrene-Butadienestyrene)^36^. In comparison with previous research, this sensor exhibits really high sensitivity with balanced sensing range for torsion and broad sensing range in the case of stretching and bending.

**Table 2:** Comparison between PDMS/rGO/PDMS structure with previously reported Graphene strain sensors.

Materials	Sensing range (strain%)	Senstivity∕gauge factor (G.F)	References
GO/PI	0.003%	54.2	^11^
Graphene assembled film	0.8–2.8%	1.934	^37^
Mulberry paper graphene	0.587%	3.82	^22^
3-nm thick graphene platelets	0–0.67%	1–33	^38^
Graphene nanowalls (GNWs)	4%	8.6 × ${10}^{4}$	^39^
GO nano-platelets	5%	30	^40^
rGO coated elastic fibres	5%	8.8	^20^
GO woven fabrics	> 7%	106	^41^
GO/PDMS	11.1% (torsion)	90.3	This work
3D graphene foam	15%	6.24	^24^
TPU and coupling of AGNW with rGO	0–15%	> 400	^42^
3D porous rGO	0.24–70%	1686.48	^43^
SBS/FLG	100%	2546	^36^
SBS graphene fibre	100%	10,083.98	^44^
GO/PDMS	130% (bending)	3.5	This work
GO/PDMS	140% (stretching)	12.12	This work

This sensor shows very good results for stretching, bending and torsion when considering both sensitivity and sensing range.

## Conclusion

Flexible strain sensors were fabricated using laser-scribed graphene oxide on PDMS and tested using a locally developed testing rig. Six degrees of freedom was achieved by using PDMS material as base, which supports sensing ranges of 140%, 130% and 11.12% for stretching, bending and torsion respectively. GFs are reported to be 12.1, 3.5 and 90.3 for stretching, bending and torsion, respectively. Sensitivity was improved by using a specific fabrication method whereby liquid PDMS was poured on rGO & GO and subsequently encapsulated with another PDMS layer. Encapsulation also helps to avoid buckling, any material damage and prevents from degrading the material as well. This sensor works for more than 1000 cycles while maintaining the hysteresis. Response time of ~42 ms and recovery time of ~78 ms was observed. Experiments also show that the response time and sensitivity is affected by sensing speed of 83.35 μm/s and 900 μm/s. Change in resistance is high at a higher speed than a lower speed. The sensor also exhibits excellent sensitivity when applied to the human wrist and finger to measure muscle motion. The sensor shows a visible difference between normal and heavy breathing after attaching to the human wrist. These sensors have significant potential in healthcare devices and wearable electronics.

## Supplementary Information

Supplementary Video 1.

Supplementary Information 1.
