# Supplementary material for: A flexible piezoresistive strain sensor based on laser scribed graphene oxide on polydimethylsiloxane
Source: Sci Rep. 2022 Mar 22;12:4882. doi: 10.1038/s41598-022-08801-0 (PMC8941115; doi:10.1038/s41598-022-08801-0)
Supplement: Supplementary file 2 — Supplementary Information 1. [file 41598_2022_8801_MOESM2_ESM.docx]

**A Flexible Piezoresistive Strain Sensor Based on Laser Scribed Graphene Oxide on Polydimethylsiloxane**

Maham Iqra^1,**^, Furqan Anwar^1^, Rahim Jan^1^, Mohammad Ali Mohammad^1,*^

1 School of Chemical and Materials Engineering, National University of Sciences and Technology, Islamabad, 44000, Pakistan.

^*^Corresponding Author: [dr.ali@nust.edu.pk](mailto:dr.ali@nust.edu.pk)

^**^ mahamiqra123@gmail.com

SUPPLEMENTARY INFORMATION:

This document contains the following supplementary data:

Figure S1 – Laser scribing system and laser characterization.

Figure S2: Visual impression of different stretching modes including stretching, bending, and torsion.

Figure S3 – Resistance vs. strain behaviour of eight (8) devices after stretching.

Figure S4 – Resistance vs. strain behaviour of twelve (12) devices after torsion.

Figure S5 – Resistance vs. strain behaviour of seven (7) devices after bending.

Figure S6 – Sensor array (2 × 2) structure.

Video S1: Change is resistance with respect to time upon bending of the hand.

Laser scriber is used to pattern the strain sensor. It has a stage used to hold the structure, which is then scribed by the laser to form the functional parts of the strain sensor. The stage can be moved in two axes to adjust the sensor. Intensity and time of pulse can be controlled by laptop using its software. Laser characterization was also performed by the National Centre of Physics.


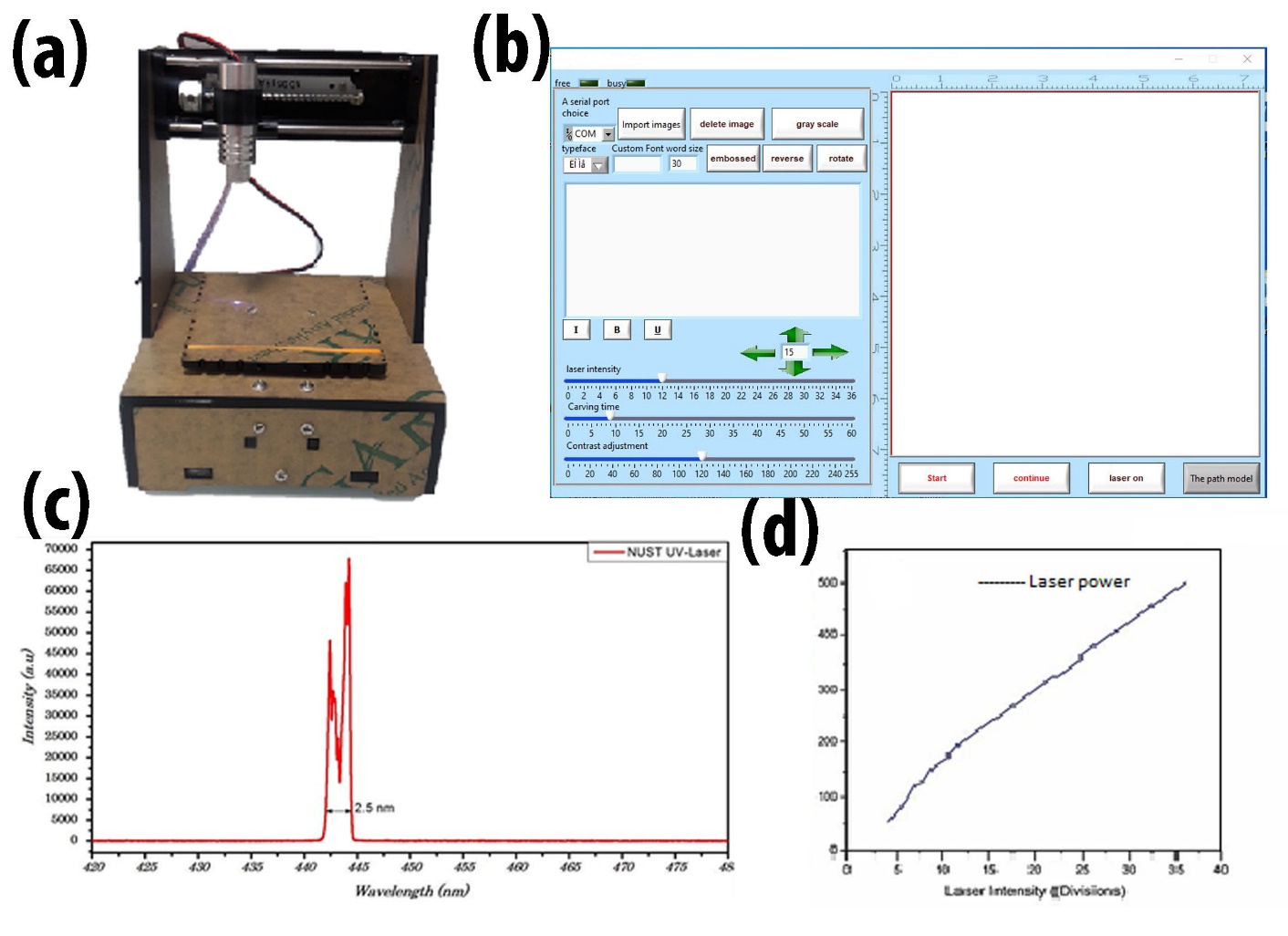


Figure S1: (a) Laser scriber used to pattern the sensor. (b) Laser software that is used to operate the laser scriber. (c) A graph between intensity and wavelength which also tells us the pointer width. (d) Laser power graph with respect to divisions shown in the software.


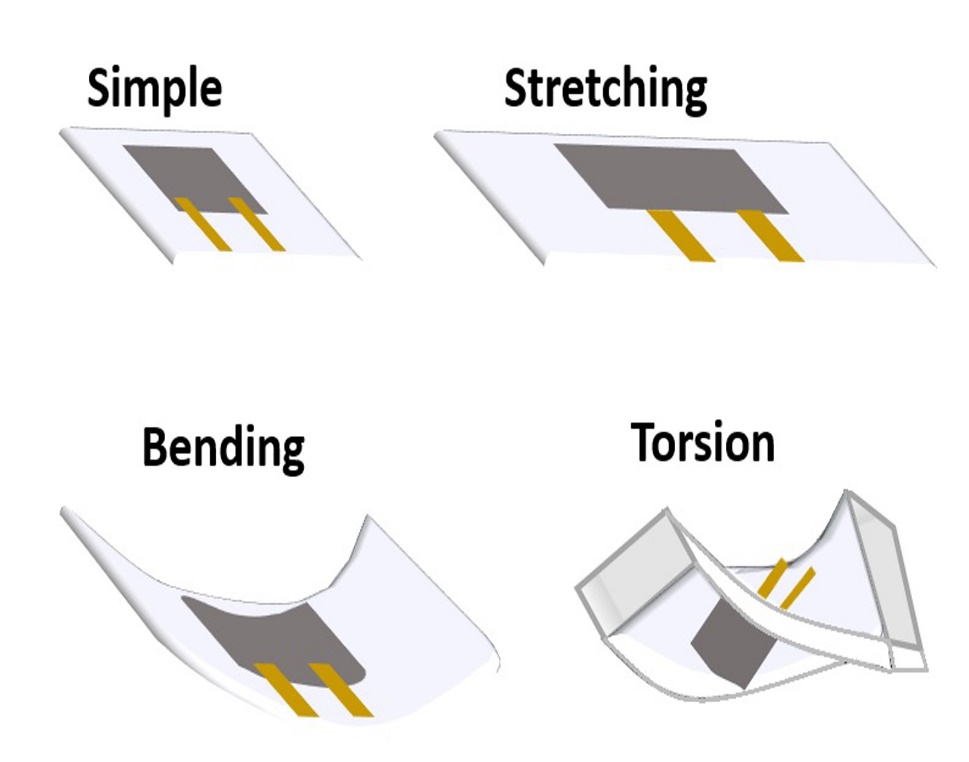


Figure S2: Visual impression of different stretching modes including stretching, bending, and torsion.

Stretching tests were performed on locally developed testing rig. Eight of the best devices among others were put to test and each device shows a similar behaviour. There is a linear increase at the start and towards the end there is the sudden increase because of cracks production after stretching.


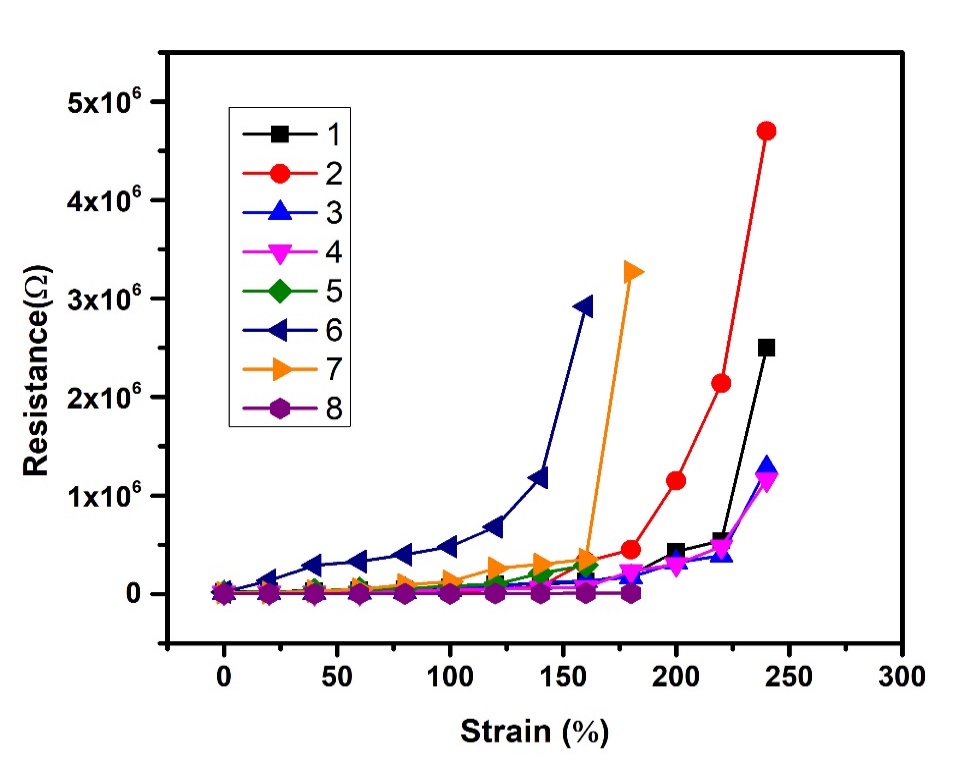


Figure S3: Resistance vs. strain behaviour of eight (8) devices after stretching.

Torsion test was performed on 12 devices. There is a linear behaviour at the start and the resistance change is very sudden after cracks formation under torsion.


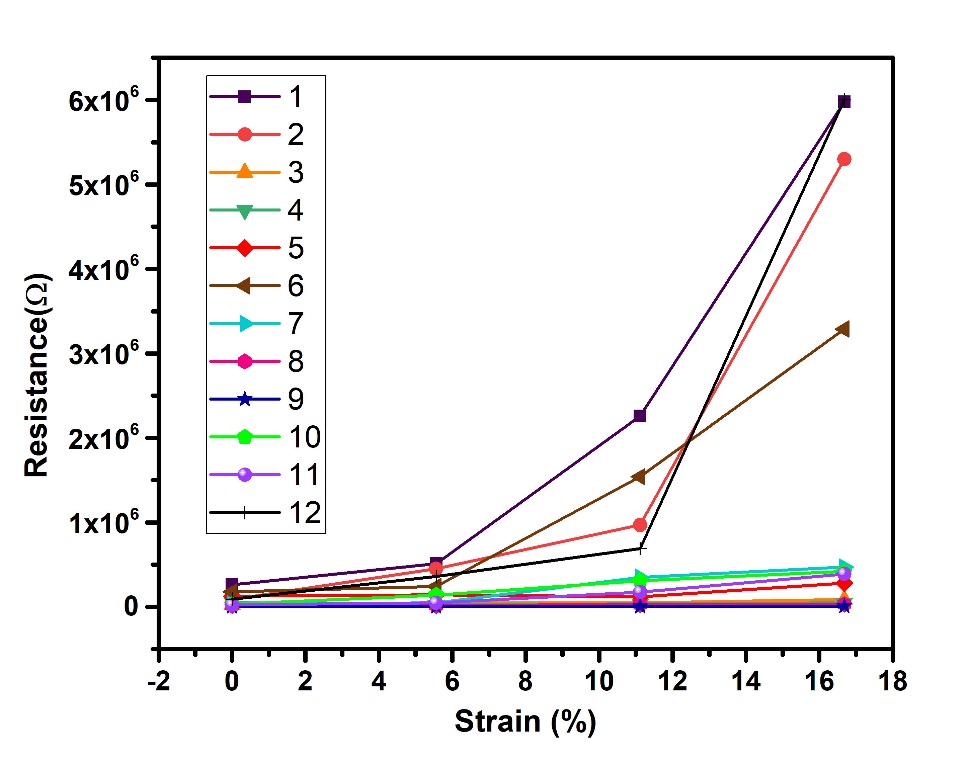


Figure S4: Resistance vs. strain behaviour of twelve (12) devices after torsion.

Bending tests were performed using the same test rig. Each bending test yields a linear behaviour throughout the sensing range.


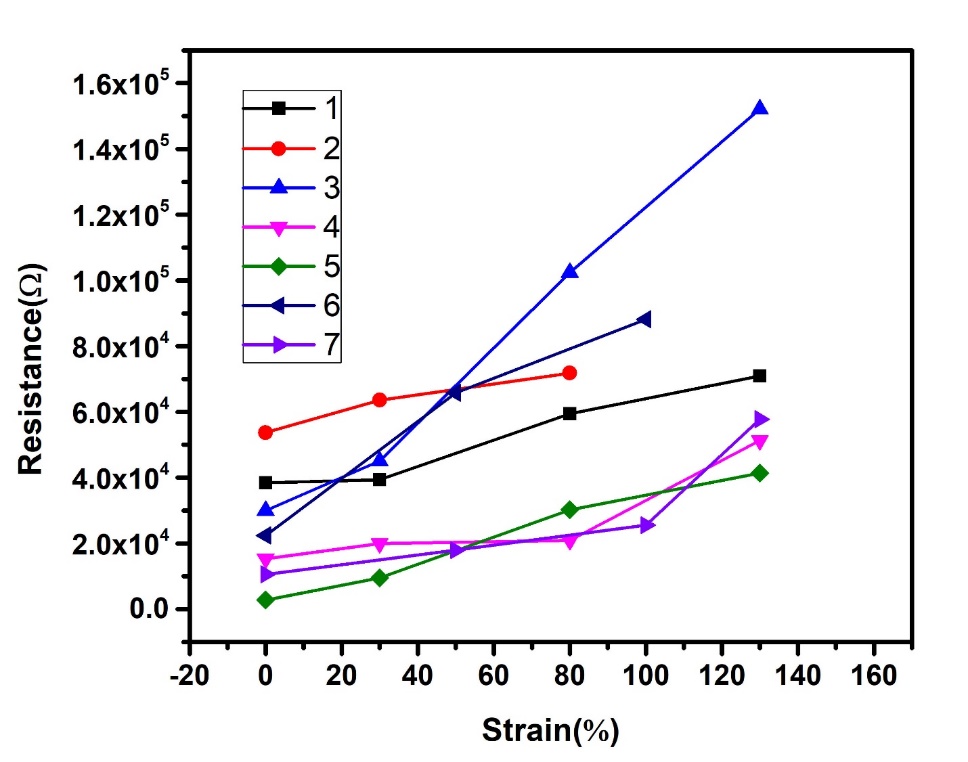


Figure S5: Resistance vs. strain behaviour of seven (7) devices after bending.

The difference of starting point in resistance is due to the difference in flakes structure and their overlapping area that cause resistance to change.


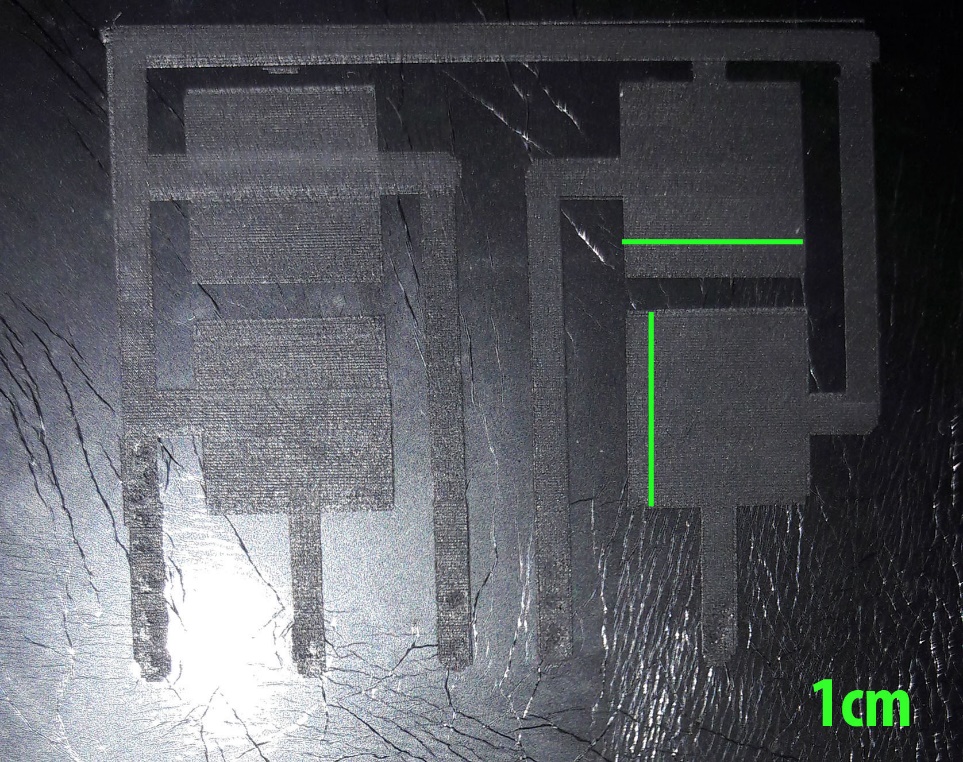


Fig S6: sensor array structure fabricated by using laser engraver where 2 × 2 strain sensor was connected to measure pressure distribution.

Video S1: Change is resistance with respect to time upon bending of the hand.
